# Expanded Carrier Screening in Chinese Population – A Survey on Views and Acceptance of Pregnant and Non-Pregnant Women

**DOI:** 10.3389/fgene.2020.594091

**Published:** 2020-11-16

**Authors:** Hiu Yee Heidi Cheng, Grace Ching Yin Wong, Yuen-Kwong Kelvin Chan, Chin Peng Lee, Mary Hoi Yin Tang, Ernest Hung-Yu Ng, Anita Sik-Yau Kan

**Affiliations:** ^1^Department of Obstetrics and Gynaecology, Queen Mary Hospital, Pok Fu Lam, Hong Kong; ^2^The Family Planning Association of Hong Kong, Wan Chai, Hong Kong; ^3^Tsan Yuk Hospital, Sai Ying Pun, Hong Kong; ^4^Department of Obstetrics and Gynaecology, The University of Hong Kong, Pok Fu Lam, Hong Kong

**Keywords:** expanded carrier screening, Chinese, pregnancy, survey, acceptance

## Abstract

**Objective:**

Recessive genetic diseases impose physical and psychological impacts to both newborns and parents who may not be aware of being carriers. Expanded carrier screening (ECS) allows screening for multiple genetic conditions at the same time. Whether or not such non-targeted panethnic approach of genetic carrier screening should replace the conventional targeted approach remains controversial. There is limited data on view and acceptance of ECS in general population, as well as the optimal timing of offering ECS to women. This study assesses views and acceptance of ECS in both pregnant women and non-pregnant women seeking fertility counseling or checkup and their reasons for accepting or declining ECS.

**Materials and methods:**

This is a questionnaire survey with ECS information in the form of pamphlets distributed from December 2016 to end of 2018. Women were recruited from the antenatal clinics and the assisted reproductive unit at the Department of Obstetrics and Gynaecology, Queen Mary Hospital and the prepregnancy counseling clinic at the Family Planning Association of Hong Kong.

**Results:**

A total of 923 women were recruited: 623 pregnant women and 300 non-pregnant women. There were significantly more non-pregnant women accepting ECS compared to pregnant women (70.7% vs. 61.2%). Eight hundred and sixty-eight (94%) women perceived ECS as at least as effective as or superior to traditional targeted screening. Significantly more pregnant women have heard about ECS compared with non-pregnant women (42.4% vs. 32.3%, *P* = 0.0197). Majority of women showed lack of understanding about ECS despite reading pamphlets that were given to them prior to filling in the questionnaires. Cost of ECS was a major reason for declining ECS, 28% (*n* = 256). Significantly more pregnant women worried about anxiety caused by ECS compared with the non-pregnant group (21.1% vs. 7.4%, *P* = 0.0006).

**Conclusion:**

Our study demonstrates that expanded carrier screening was perceived as a better screening by most women. Prepregnancy ECS maybe a better approach than ECS during pregnancy, as it allows more reproductive options and may cause less anxiety. Nevertheless, implementation of universal panethnic ECS will need more patient education, ways to reduce anxiety, and consensus on optimal timing in offering ECS.

## Introduction

Recessive genetic diseases impose physical and psychological impacts on both newborns and parents. These parents are often not aware of being carriers, and unexpected birth of affected babies will strike them hard and may lead to delayed diagnoses or delayed treatment for the babies.

The risk of having a newborn with an autosomal recessive disorder was estimated to range from 1.7 to 30 in one thousand neonates (Baird et al., 1988; United Nations Scientific Committee on the Effects of Atomic Radiation, 1994; Sankaranarayanan, 1998), and foreseeably will further increase as those with diseases are living longer as technology advances. Different ethnicity accounts for the wide range of prevalence. For example, cystic fibrosis (CF), which occurs in 1 in 2000–3000 births in white population with a carrier frequency of 1 in 29 individuals (Modaresi et al., 2017), is rarely seen in Chinese and African populations. Tay-Sachs disease, a recessive lipid storage disease, occurring in 1 in 3500 Ashkenazi Jews (Rozenberg and Pereira, 2001) is occurring only in 1 in 320,000 newborns in the United States (David, 2012). Prevalence of α-thalassemia and β-thalassemia carrier is 7.8% and 2.2%, respectively, in Chinese population (Lai et al., 2017).

Development of cutting-edge technologies with reduction of costs for DNA analysis and sequencing has resulted in rapid evolvement of genetic testing technologies, such as next generation sequencing (NGS). Panel-based high throughput NGS can screen for more than 600 genetic conditions. This screening test is termed expanded carrier screening (ECS). Estimating prevalence of carrier couples of autosomal recessive disorders in Chinese population with ECS is difficult, as this depends on the number of diseases included in different ECS panels. Zhao et al. (2019) reported that 29% of the carrier couples identified through their ECS panel would not have been identified through the existing traditional targeted screening, which mainly screened for thalassemia carrier couples. In another retrospective modeling analysis in the United States (Haque et al., 2016), two professional guidelines-based screening panels modeled 55.2 hypothetical fetuses affected per 100 000, whereas ECS modeled 159.2 fetuses per 100 000. The difference between the prevalence estimated by ECS is likely due to the different number of genetic diseases included in the two panels, with the former study including 11 genetic diseases while the latter study included 94 genetic conditions.

In view of the wide coverage of genetic diseases, ECS has attracted attention from consumers and healthcare workers. However, whether a non-targeted pan ethnic universal screening should replace the conventional targeted or ethnic-based approach carrier screening remains controversial (Langlois et al., 2015). While its wide coverage of genetic disease attracts a lot of attention, there are debates on the genetic diseases to be included, and ethics on imposing unnecessary anxiety on clients who may not be adequately counseled.

There is currently limited data on the views and acceptance of ECS in the Chinese population. The aim of our study is to assess the knowledge and acceptance of ECS by pregnant and non-pregnant women in Hong Kong, in order to bridge the transition and identify gaps in replacing conventional ethnic-based screening with panethnic universal screening.

## Materials and Methods

This is a cross-sectional questionnaire study approved by the Institutional Review Board, The University of Hong Kong/Hospital Authority Hong Kong West Cluster (trial registration number UW16-230), as well as the Health Services Subcommittee of the Family Planning Association (Trial registration number OA1-2). Chinese women aged 18 or above were recruited from July 2016 to the end of 2018 from the antenatal clinics and the assisted reproductive unit at Department of Obstetrics and Gynaecology, Queen Mary Hospital and the prepregnancy counseling clinic at the Family Planning Association of Hong Kong. Those who could not read or understand Chinese or English were excluded.

During the medical consultations, couples were given the pamphlets with ECS information and a self-administered questionnaire survey after written informed consents were taken. The questionnaire surveys were collected before leaving the clinic or at subsequent visits. Therefore, the patients had at least 2 h before filling in the questionnaire. Some patients will submit their questionnaires upon their next visits, which would be a few weeks later. The questionnaire included questions on whether they had heard of ECS, their first impression on ECS compared to the conventional ethnic-targeted screening, how they felt about undertaking ECS, whether they will or will not accept to receive ECS and their reasons, and their basic demographics (please refer to Supplementary Material). The short form of the Spielberger State-Trait Anxiety Inventory (STAI-6) was used in measuring the anxiety of pregnant compared with non-pregnant women. STAI-6 scores less than 40 are considered low anxiety while STAI-6 scores higher than 40 are considered women with high anxiety (Marteau and Bekker, 1992).

The primary outcome was patients’ acceptance and literacy on ECS, as well as the factors affecting the choice of ECS in prepregnancy and pregnant patients. Secondary outcomes included anxiety caused by ECS. The basic demographics of participants were compared. The data were analyzed using SPSS 25.0. For quantitative variables, descriptive statistics were calculated and choices between pregnant and non-pregnant women were compared using Chi-square test.

## Results

### Basic Demographics and Background of Respondents

Nine hundred and twenty-three women were recruited and completed the questionnaire: 623 (67.5%) pregnant and 300 (32.5%) were non-pregnant. Demographic data and characteristics of women between pregnant and non-pregnant group were compared in Table 1. In our cohort, the pregnant women are significantly younger than the non-pregnant women. The mean gestational age of pregnant women was 14 weeks and 2 days of gestation. Comparing between pregnant and non-pregnant groups, there was no significant difference seen in their previous obstetric history, education level and monthly salary income. There were significantly more working patients and more patients without a religion in the non-pregnant group.

**TABLE 1:** Demographic data and characteristics of women (*n* = 923).

	**Non-pregnant women (*n* = 300)**	**Pregnant women (*n* = 623)**	***P*-value**
**Age (years)**			
Below 20	0 (0)	6 (1.0)	< 0.0001
20–25	13 (4.3)	33 (5.3)	
26–30	29 (9.7)	148 (23.8)	
31–35	103 (34.3)	283 (45.4)	
36–40	139 (46.3)	138 (22.2)	
41 or above	16 (5.3)	15 (2.4)	
*Mean gestational age*	NA	14 weeks 2 days ± 8 SD	NA
**Previously carried a pregnancy with an abnormal fetus**			
Never	286 (95.3)	601 (96.5)	0.5421
Once	14 (4.7)	20 (3.2)	
Twice	0 (0)	0 (0)	
Three times or more	0 (0)	2 (0.3)	
**Education level**			
Below primary school	3 (1.0)	3 (0.5)	0.5613
Secondary or matriculation	96 (32.0)	216 (34.7)	
University graduate or above	201 (67.0)	404 (64.8)	
**Occupation background**			
Full-time	249 (83.0)	452 (72.6)	0.0015
Part-time	13 (4.3)	35 (5.6)	
Unemployed/Housewife	38 (12.7)	136 (21.9)	
**Religious belief**			
No religion	247 (82.3)	416 (66.8)	< 0.0001
Christian	28 (9.3)	102 (16.4)	
Catholic	16 (5.3)	58 (9.3)	
Buddhist	4 (1.3)	33 (5.3)	
Muslim	2 (0.7)	2 (0.3)	
Others	3 (1.0)	12 (1.9)	
**Monthly family income (HKD)**			
Below $10,000	4 (1.3)	40 (6.4)	0.0791
10,001 to $30,000	55 (18.3)	132 (21.2)	
30,001 to $50,000	91 (30.3)	170 (27.3)	
50,001 to $70,000	76 (25.3)	104 (16.7)	
70,001 or above	74 (24.7)	177 (28.4)	

### Impression and Knowledge of ECS Among Pregnant and Non-Pregnant Women

Most of the pregnant women were in their second trimester upon recruitment for the questionnaires. The impression of ECS among pregnant and non-pregnant women is listed in Table 2. Among the 623 pregnant women, 306 (49.1%) had never heard of ECS, 235 (37.7%) women heard of it before but did not fully understand the nature of the test, and only 29 (4.7%) women had heard of it before and fully understood about ECS. Among the 300 non-pregnant women, 172 (57.3%) women had never heard of ECS, 86 (28.7%) women heard of it before but did not fully understand the nature of ECS, and only 11 (3.7%) women had heard of it before and understood about ECS. Significantly more pregnant women have heard of ECS compared with non-pregnant women (*p* value = 0.0197). There was missing data in 84 women in various questions on the questionnaires. Women were asked about their first impression of ECS compared to conventional targeted screening. Among the 300 non-pregnant women, 253 (84.3%) had the impression that ECS was better than conventional screening, whereas only 469 women (75.3%) in the pregnant group thought ECS was better than conventional screening. Seven pregnant women (7/623 = 1.1%) had the impression that ECS is worse than conventional targeted screening, compared with one woman (0.3%) in the non-pregnant group. Forty-seven women did not indicate their impression on the questionnaire.

**TABLE 2:** Impression of expanded carrier screening.

	**Pregnant women *n* = 623 (%)**	**Non-pregnant women *n* = 300 (%)**	***P*-Value**
**Have you ever heard of expanded carrier screening?**			
Never heard of it	306 (49.1)	172 (57.3)	0.0197
Heard before but did not fully understand nature of the test	235 (37.7)	86 (28.7)	
Heard of it before and understand about ECS	29 (4.7)	11 (3.7)	
**What is your first impression of expanded carrier screening compared to conventional targeted screening?**			
Better than conventional targeted screening	469 (75.3)	253 (84.3)	0.0104
As good as conventional targeted screening	112 (18.0)	34 (11.3)	
Worse than conventional targeted screening	7 (1.1)	1 (0.3)	

Specific questions on knowledge of ECS were set up in the questionnaires as listed in Table 3. Overall the percentage of getting correct answers from women after reading the pamphlets ranged only from 38.0% to 55.5%. There was no significant difference between pregnant and non-pregnant group.

**TABLE 3:** Knowledge of expanded carrier screening.

**Question**	**Pregnant women with correct answer *n* = 623 (%)**	**Non-pregnant women with correct answer *n* = 300 (%)**	***P*-value**
ECS allows screening for more than 100 genetic disorders by one test.	346 (55.5)	152 (50.7)	0.389
Depending on the condition tested, an abnormal test result for myself means I am a carrier of the disease.	273 (43.8)	147 (49.0)	
Depending on the condition tested, an abnormal test result for myself means I will develop the disease	320 (51.4)	146 (48.7)	
A normal test result for myself means my future offspring will never have the disease tested by the panel	277 (44.5)	114 (38.0)	
When both my partner and myself have abnormal result on the same condition which is of autosomal recessive inheritance, there is a 25% chance that our future child will have the disease caused by the gene	288 (46.2)	144 (48.0)	

### Women Hypothetically Chose ECS and Factors Influencing the Decision

Reasons for undertaking or declining ECS under hypothetical scenario are listed in Table 4. Comparing pregnant and non-pregnant women, significantly more non-pregnant women opted for ECS (70.7% vs. 61.2%, *P*-value 0.0047). The main reason in these women was to have information guiding them to make informed choices or preventing them to have an affected baby.

**TABLE 4:** Reasons for undertaking or declining ECS under hypothetical scenario.

	**Number (%) of pregnant women**	**Number (%) of non-pregnant women**	***P*-value**
**Reasons for choosing ECS by pregnant women (n = 381) and non-pregnant women (n = 212) (can choose more than one option)**			
It determines the risk of me and my partner having a child with a recessive disorder, and hence facilitates meaningful reproductive choices	272 (71.4)	184 (86.9)	0.0002
It could help prevent the birth of affected children when both I and my partner are known to be carriers	144 (37.8)	107 (50.5)	0.0028
It could contribute to early therapeutic procedures in the neonatal period when both I and my partner are known to be carriers	175 (45.9)	89 (42.0)	0.3535
Others	11 (2.9)	3 (1.4)	0.2578
**Reasons for NOT choosing ECS by pregnant women (n = 355) and non-pregnant women (n = 121) (can choose more than one option)**			
It is too new to me	45 (12.7)	6 (5.0)	0.0178
It cannot detect all carriers of the conditions tested	18 (5.1)	6 (5.0)	0.961
I want to have information on other conditions	24 (6.8)	9 (7.4)	0.0642
I do not want information related to carrier status	9 (2.5)	0 (0)	0.254
I would like to have the test only when I become pregnant	34 (9.6)	33 (27.3)	0.0003
It may lead to anxiety when I or my partner is tested to be carriers	75 (21.1)	9 (7.4)	0.0006
I cannot afford this expensive test	185 (52.1)	73 (60.3)	0.117
Others	72 (20.3)	15 (12.4)	0.053

Among the pregnant women, 381 would like to undertake ECS. Two hundred and seventy-two (71.4%) women thought ECS could help to confirm whether their future children are at risk of recessive genetic diseases, thereby guiding them to make informed choices. Another 144 (37.8%) women would like to know if her partner and herself were carriers of recessive genetic disease and thought this could help to prevent them from having an affected baby. One hundred and seventy-five (45.9%) pregnant women thought knowing whether themselves and their partners are carriers could help her affected baby to receive treatment earlier.

Among the non-pregnant women, 212 women opted ECS. One hundred and eighty-four (86.9%) women thought ECS could help to confirm whether their future children were at risk of recessive genetic diseases, thereby guiding them to make informed choices. Another 107 (50.5%) women would like to know if her partner and herself were carriers of recessive genetic disease and thought this could help to prevent them from having an affected baby. Eighty-nine (42%) pregnant women thought knowing themselves and their partners are carrier could help her affected baby to receive treatment earlier.

### Women Choosing Not to Undergo ECS and Factors Influencing Decision

Comparing pregnant and non-pregnant women, significantly more pregnant women opted not to undertake ECS (57.0% vs. 40.3%, *P*-value = 0.00005) under the hypothetical scenario that one ECS test costs HKD$4000 per person. Among these women, most of them opted not for ECS because they cannot afford this expensive test. Other reasons included the anxiety ECS would cause and that they were not prepared to accept a new test.

Among the pregnant women, 185 (52.1%) women thought ECS was too expensive. Seventy-five (21.1%) women worried that the result of ECS would make them feel anxious. Forty-five (12.7%) women were not prepared to accept a new test. Among the non-pregnant women, 33 women (27.3%) preferred to have the test only when they became pregnant. Seventy-three (60.3%) women thought ECS was too expensive, and 9 (7.4%) women thought the result of ECS would make them feel anxious. Only 6 women (5%) were not prepared to accept a new test. State-Trait Anxiety Inventory (STAI) scores were compared between pregnant and non-pregnant women. Non-pregnant women had a higher mean STAI score when compared with pregnant women (42.0 vs. 37.7 respectively, *P*-value = 0.150). As women could be more anxious before getting pregnant, this may account for the paradoxical response with non-pregnant women having higher STAI scores.

## Discussion

Panethnic non-targeted ECS is gradually gaining popularity, over traditional non-targeted screening, in view of the increasing multiracial society. In Hong Kong, it is a voluntary and self-financed screening provided by the private sector. Many professional societies have published statements with regards to ECS in the past few years. The American College of Obstetricians and Gynecologists (ACOG), the American College of Medical Genetics and Genomics (ACMG) and the National Society of Genetic Counselors (NSGC) published a joint statement in 2015 stating the acceptability of ECS as a carrier screening test, on the other hand emphasizing its complexities in choice of disease to be screened, as well as pre- and post-test counseling (Edwards et al., 2015). ACOG published another individual committee opinion in 2017 stating both ethnic-specific, panethnic, and expanded carrier screenings are all acceptable strategies for prenatal or pre-pregnancy carrier screening (American College of Obstetricians and Gynecologists, 2017). Despite different societies supporting the launching of ECS, there are still a number of hurdles that one has to overcome, which includes issues with pre- and post-test counseling, best timing of carrying out ECS, and the possibility of anxiety caused due to the time required to wait for test results. This study aims to explore acceptability of ECS and the reasons of opting in or opting out ECS among pregnant and non-pregnant women.

This study showed that significantly more pregnant women had heard about ECS compared with the non-pregnant group (42.4% vs. 32.4%, *P* = 0.0197), although the numbers of women who heard and fully understood ECS were limited. One of the reasons could be that the pregnant women were more prepared to undergo different screenings aiming to lead to a healthy baby, compared with the non-pregnant women who may just have started to think about their reproduction options. For similar reasons, pregnant women were more cautious with ECS than traditional targeted screening compared with non-pregnant women, as reflected by a significantly smaller percentage of pregnant women thought that ECS was better than traditional targeted screening compared with non-pregnant women (75.3 vs. 84.3%, *P* = 0.0104).

In our cohort, respondents have generally poor knowledge of ECS despite reading information pamphlets prior to answering questionnaires. In contrast, Schuurmans et al. counseled 119 couple individually by trained general practitioners who were assessed by genetic professionals to be competent in carrying out pre-test counseling. Majority of the patients (97%) were identified as having sufficient knowledge to make informed decisions (Schuurmans et al., 2019). This could be due to the complexity of ECS and less favorable form of information delivery with pamphlet in our cohort. Devising methods to deliver information to patients in a manner that can effectively reduce anxiety is essential to help them psychologically prepare for the results. Both ACMG and ACOG supported verbal, audio-visual, pamphlets form of pretest counseling (Edwards et al., 2015; American College of Obstetricians and Gynecologists, 2017). A study interviewing geneticists in Europe also supported the use of audiovisual aids instead of detailed pre-test counseling, to avoid overloading patients (Janssens et al., 2017). Up to date, there is no consensus on the method of information delivery with regards to offering ECS.

Timing of offering ECS has always been arbitrary. In our study, significantly more non-pregnant women accepted ECS hypothetically compared to pregnant women (70.7% vs. 61.2%). This finding is consistent with a previous retrospective study comparing ECS actual uptake rate between pregnant and non-pregnant women. The study found significantly more non-pregnant women accepting ECS after pre-test counseling by geneticists compared to pregnant women in their 13–14 weeks of gestation (Larsen et al., 2019). When it comes to the reason for electing or declining ECS, the top reason in both groups for electing ECS was that women thought ECS is helpful in confirming whether their children were at risk of recessive genetic diseases, thereby guiding them to make informed choices. This finding is consistent with the previous study published by Propst et al. (2018) on perspectives of pregnant women on ECS. In our cohort, it seems more non-pregnant women viewed this as the top reason for selecting ECS compared with pregnant women (86.9% vs. 71.4%). Our postulation was that some pregnant women may choose not to terminate pregnancies for fetal abnormalities because of ethical reasons. Moreover, more women in the non-pregnant group wished to have ECS as they thought it could help them from having an affected baby, compared with the pregnant group. These responses highlighted that preconception period would be a better timing for undergoing ECS, as preconception testing allows more time for pre- and post-test counseling, and more reproductive options for carrier couples, including preimplantation genetic testing through *in vitro* fertilization.

Anxiety for both patients and healthcare workers caused by ECS is another hurdle in launching universal ECS. Offering ECS in the preconception instead of prenatal period may reduce anxiety caused by pressurized time constraint for counseling and testing in pregnancy. In Hong Kong, the legal limit for termination of pregnancy is 24 weeks of gestation. This limit sets psychological pressure to women in terms of time preparing to undergo the test, time required to generate the test, and time required in making informed decisions once the couple are diagnosed to be carriers. In our cohort, more pregnant women declined ECS because of anxiety compared with non-pregnant women. This illustrates the likelihood of anxiety generated by ECS with limited time frame for prenatal counseling and testing, if launched in the prenatal period.

Time pressure also sets burden on healthcare workers in carrying out pre- and post-test counseling, as well as pressure on knowledge as ECS with NGS is a relatively new technology which included a multitude of recessive genetic diseases which are rare. Lynch et al reported that a median time of 64 minutes was required per women in providing post-test counseling who were screened positive as a carrier of at least one genetic condition (Lynch et al., 2018). As a general practice in Hong Kong, prepregnancy and prenatal genetic counseling for at risk couples are mostly performed by Maternal Fetal Medicine Subspecialists or obstetrician-gynecologists with special interest in prenatal diagnosis and reproductive genetics. For complex conditions, referral to clinical geneticists can be arranged. For the general population, prepregnancy counseling will be carried out by general practitioners. With universal launching of ECS, there will be an expected rise in demand on both obstetricians and geneticists’ expertise in counseling. Time required for pre- and post-test counseling is enormous if there is increased ECS uptake. There is also pressure on TAT of ECS reports, so concomitant testing is preferred. Also clinician needs to find a genetic laboratory for prenatal genetic diagnosis for at risk couple too.

Cost of the test is another reason for not accepting ECS in our cohort. A retrospective review found an increase of ECS uptake rate from 3.3% to 17.5% following the decrease in out-of-pocket cost of ECS from USD$350 to $99 amongst infertile couples. The relatively low uptake rate in this study could be due to the lack of advertisement of ECS back in 2013 during the study period (Higgins et al., 2015). The author also suggested women with infertility are a special group which may have a higher uptake rate due to their health-seeking behavior and motivation. Clarke et al carried out a survey on willingness to pay for genome sequencing reporting carrier results for genetic conditions (Clarke et al., 2018), and reported association between income level with willingness to pay. Apart from willingness to pay, whether ECS is cost-effective is still debatable. Cost-effectiveness analysis was carried out in a survey study by Beauchamp et al., which showed relative to minimal screening, preconception ECS reduces the affected birth rate and is estimated to be cost-effective (Beauchamp et al., 2019). However, cost-effectiveness of ECS depends on the number of and the kinds of genetic conditions to include, as well as whether to compare with minimal screening or traditional targeted screening. In our cohort, cost-effectiveness was not analyzed. However, 256 (29%) expressed concerns on the cost of ECS and this was the major reason for declining ECS if they had to pay HKD4000 for one test. With current cost of ECS, it would be difficult for government to support free offering of ECS. However, with rapid development of genetic testing technology, it is foreseeable that the cost of ECS will fall dramatically in the near future.

To date, our study is the largest questionnaire study of both pregnant and non-pregnant women, with total of 923 women recruited. Our study had the advantage of recruiting patients from one of the two large tertiary hospitals in Hong Kong, responsible for over 3500 deliveries per year. It involves recruitment of both pregnant and non-pregnant women, as well as infertile couples and couples without infertility attending for prepregnancy checkup. Therefore, the results can represent both categories of women. Briggs et al reported up to 51% women with no desire for testing and 28% women would not accept ECS if they had to pay out of their pockets (Briggs et al., 2017). Our study reported a higher ECS uptake rate, possibly because of the different ethnic groups between the two studies. One limitation of our study is that the acceptance of ECS is only hypothetical, and the decision may change when women actually have to decide on whether to undertake ECS in a real clinical setting. The current study demonstrates the deficient knowledge on ECS in the general population, and that pamphlet illustration of ECS may not be the best method in delivering information for such complex screening. Nonetheless, this study clearly demonstrates the need for further education of public prior to implementation of universal panethnic ECS to bridge the gap.

## Conclusion

Expanded carrier screening is likely to be widely taken up by consumers due to its wide coverage of genetic diseases and lowering of cost as technology advances. To implement ECS responsibly, it is important to overcome several hurdles, such as patient education, cost of ECS, ways to reduce anxiety, diseases to be included in panels and consensus on optimal timing in offering ECS. Knowing patient’s impressions and attitudes toward ECS is a crucial step in overcoming the above hurdles. Concerning the optimal timing, prepregnancy ECS maybe a more reasonable approach. This can allow more reproductive options including preimplantation genetic testing as well as reduction in anxiety if the couples are screened carriers, compared with when they are screened to be carriers during pregnancies. However, further studies are still required prior to the consensus of optimal timing and responsible launching of universal ECS to the public.

## Data Availability Statement

The raw data supporting the conclusions of this article will be made available by the authors, without undue reservation.

## Ethics Statement

The studies involving human participants were reviewed and approved by The University of Hong Kong/Hospital Authority Hong Kong West Cluster and Health Services Subcommittee of the Family Planning Association. The patients/participants provided their written informed consent to participate in this study.

## Author Contributions

HC is the corresponding author and is responsible for analyzing the data, writing up the manuscript and the submission of the manuscript. GW helped to recruit patients in the Family Planning Association (FPA) and helped to overlook the FPA study site. Y-KC was responsible for manuscript review and advice on study design. CL, MT, and EN provided expert advice, and helped to recruit participants and to revise the manuscript. AK involved in the conception and design of the study, recruitment of participants, and final revision of the abstract and manuscript to be submitted for review. All authors contributed to the article and approved the submitted version.

## Conflict of Interest

The authors declare that the research was conducted in the absence of any commercial or financial relationships that could be construed as a potential conflict of interest.
