# Supplementary material for: Expanded Carrier Screening in Chinese Population – A Survey on Views and Acceptance of Pregnant and Non-Pregnant Women
Source: Front Genet. 2020 Nov 16;11:594091. doi: 10.3389/fgene.2020.594091 (PMC7701308; doi:10.3389/fgene.2020.594091)
Supplement: Supplementary file 1 [file Data_Sheet_1.PDF]

DEPARTMENT OF OBSTETRICS AND GYNAECOLOGY
THE UNIVERSITY OF HONG KONG

INFORMATION SHEET ON EXPANDED CARRIER SCREENING

What is "expanded carrier screening"?

- High-throughput genotyping and sequencing approaches allow for efficient screening of a large number of conditions simultaneously. Use of this technology provides information regarding many more conditions than the currently recommended screening guidelines and is referred to as expanded carrier screening.
- This test analyses specific changes in the DNA called mutations. Certain mutations can make it more likely that they could pass on a hereditary condition through a pregnancy, and the child will be at risk to be affected.

What are the objectives and benefits of "expanded carrier screening"?

- Carrier screening for inherited genetic conditions is an important component of preconception and prenatal care. Carrier identification allows for preconception planning as well as the option of prenatal diagnosis for the couple at risk. Early identification of affected pregnancies allows condition-specific counselling and care.
- The results may help couples make more informed decisions regarding their family and discover risks of their children inheriting genetic disorders and disease.
- The results may also benefit other family members. For participants who test positive, their biological relatives are more likely to test positive for the same mutation(s), thereby discovering previously unknown risks.

Who should consider having this test?

- Women and her partner of reproductive age who are planning or considering conception. Pregnant couples are less preferred because of the time pressure to receive test result. The test is voluntary. The decision to accept or decline genetic carrier screening is completely up to you.

What types of samples are required for this test?

- Saliva/swab/blood sample from women, and saliva/swab/blood sample from her partner for couple testing.

How is the test done?

- The samples shall be submitted to the test provider laboratory for DNA extraction, and for couple testing for expanded carrier screening.

How many conditions are tested?

- More than 100 genetic conditions can be screened at the same time. The majority are conditions tested are autosomal recessive. A few conditions are X-linked conditions. Some of the diseases carry a risk for intellectual disability, or lead to shortened life expectancy. Some conditions improve with early intervention while some conditions have limited or no treatment options.

When will the results be available?

- Test results are available in 6 weeks' time. If both you and your partner are being tested simultaneously, you are authorizing the release of your results to your partner, which may include sensitive genetic information.

What are the possible test results?

- Screen negative result – you/and your partner are not a carrier of any disease-causing mutation tested; this reduces the likelihood of the carrier state of the condition, but a residual risk of being a carrier always remains
- Screen positive result – you/and your partner are a carrier of variant in a particular gene linked to a particular genetic condition.
- Individuals or at risk couples who receive an abnormal result shall be referred to clinical geneticist for post-test genetic counselling

What are the limitations of the test?

- It does not analyse every mutation associated with each disease, nor does it analyse all known genetic diseases.
- This test analyses specific DNA mutations associated with genetic conditions. This test may help to reduce risk, but will not eliminate risks completely. However, as with all screening tests, there is a chance of a false positive or false negative result. A "false positive" refers to the identification of a gene mutation that is not present. A "false negative" is the failure to recognize a mutation that indeed exists.
- Some of the conditions screened for have limited or no treatment options.
- This screening test does not replace antenatal screening and prenatal diagnostic tests. Hence couples who are pregnant are still advised to go through routine antenatal screening tests and check up.

What are the other important considerations in choosing the test?

- Genetic testing may reveal sensitive information about your own health. Your results may indicate increased likelihood that other family members, such as siblings, also carry disease mutations.
